# Acknowledgement to Reviewers of *Materials* in 2016

**DOI:** 10.3390/ma10010062

**Published:** 2017-01-12

**Authors:** Materials Editorial Office

**Affiliations:** MDPI AG, St. Alban-Anlage 66, 4052 Basel, Switzerland; materials@mdpi.com

The editors of *Materials* would like to express their sincere gratitude to the following reviewers for assessing manuscripts in 2016.

We greatly appreciate the contribution of expert reviewers, which is crucial to the journal’s editorial process. We aim to recognize reviewer contributions through several mechanisms, of which the annual publication of reviewer names is one. Reviewers receive a voucher entitling them to a discount on their next MDPI publication and can download a certificate of recognition directly from our submission system. Additionally, reviewers can sign up to the service Publons (https://publons.com) to receive recognition. Of course, in these initiatives we are careful not to compromise reviewer confidentiality. Many reviewers see their work as a voluntary and often unseen part of their role as researchers. We are grateful to the time reviewers donate to our journals and the contribution they make. 

If you are interested in becoming a reviewer for *Materials*, see the link at the bottom of the webpage http://www.mdpi.com/reviewers.

The following reviewed for *Materials* in 2016:
Aalberg, ArneHerranz, FernandoPark, Joo-CheolAasmundtveit, Knut E.Herranz, JesúsPark, K.Abate, AdamHerrero-Martínez, José M.Park, Soo-JinAbbaszadeh, S.Herreros, Ángela MeladoPark, SungkyunAbdelaziz, RamzyHerzig, PeterParlett, ChristopherAbdessalem, A. BenHess, Dennis W.Parodi, FabrizioAbdul-Aziz, AliHeuler, PaulParrino, FrancescoAbenojar, J.Hibino, TakashiParsons, J.G.Aboulkhair, Nesma T.Hickok, Noreen J.Pastrana-Martinez, L.M.Abraham, Wolf-RainerHidalgo-Bastida, AraidaPatane, AmaliaAbrahams, IsaacHidalgo-Manrique, P.Patel, VikasAbraham, O.Hideto, MinamiPatenaude, MathewAbt, TobiasHierro-Rodriguez, A.Patra, AnirbanAchelle, SylvainHigashi, YuichiPatra, Jayanta KAchilli, SimonaHiggins, Michael D.Patra, NiranjanAdam, Jose M.Hill, CallumPatrick, BamonteAdamopoulos, StergiosHill, David J.T.Patsios, Sotiris I.Adolfsson, ErikHilloulin, BenoitPattanaik, HimansuAfanasiev, P.Hintikka, JoukoPatterson, Brian M.Afshinnia, KavehHirai, NobumitsuPatterson, JenniferAfzaal, MohammadHirai, ToshihiroPaul, S.Aggarwal, SrijanHirakawa, HidetadaPauli, TroyAgrawal, DineshHirohata, M.Payne, GregoryAgrela, FranciscoHo, JohnnyPeet, MathewAguey-Zinsou, K.-F.Ho, Wei-YuPei, YutaoAguiar, JoseHo, Wen-JengPelled, GadiAhangar-Asr, AlirezaHoffmeister, HansPellegrino, CarloAhmad, ZeeshanHofmeister, WilliamPelliccione, Christopher J.Ahmed Shaikh, F.U.Hohenwarter, AntonPellicer, EvaAhn, Byung TaeHolloway, JuliannePelosato, RenatoAhn, ByungminHolmberg, KennethPeng, ShengjieAili, DavidHolroyd, N.J. HenryPeng, XiaoguangAimable, AnneHolzel, ChristinaPeng, ZhiliAïssou, KarimHonciuc, AndreiPepe, MarcoAkiyama, EijiHong, Che-WunPereira, Elsa Maria VazAkkutlu, I. YucelHong, Nguyen HoaPereira De Oliveira, Luiz A.P.Alam, ParvezHong, Soon-JikPérez, Emilio M.Alam, ShahriaHong, YoungkeunPérez, GloriaAlavi, SamanHooshmand, NasrinPérez-Cadenas, Agustín F.Albano, MartaHooshmand, SalehPérez-Maqueda, Luis A.Albini, AngeloHooton, R. DougPérigo, E.A.Alegre, CinthiaHöppel, Heinz-WernerPerras, Matthew A.Alessandri, IvanoHorie, YujiPerreault, FrancoisAlessi, AntoninoHoriuchi, YuPerron, A.Alexander, Dennis R.Hort, NorbertPerrot, ArnaudAlexander, MorganHorton, RobertPerry, ChristopherAlexiadis, AlessioHossain, AnwarPersson, CeciliaAlfonso, San-MiguelHossain, ZahidPerut, FrancescaAl-Haik, MarwanHosseini, MahsaPeshek, TimothyAlhwaige, Almahdi A.Hou, ChongPetit, CamilleAli, IbrahimHoung, BoenPetrov, R.H.Ali, MajidHowell, Bob A.Pettignano, AlbertoAl-Jamal, WafaHowell, Carol A.Peyvandi, AmirpashaAlkasrawi, MalekHoying, James B.Pezzato, LucaAl-Lagtah, NasirHsi, Chi-ShiungPfeifer, CarmemAllais, FlorentHsiang, Hsing-I.Philipose, U.Allison, Ashlee M.Hsiao, Sheng-HueiPhonsri, WasineeAlMangour, BandarHsieh, Chien-KuoPichat, PierreAlmqvist, BjarneHsu, Cheng-HsunPichler, BernhardAlonso, Mar M.Hsu, Chih-ChiehPierre, AlexandreAlpatova, AllaHsu, Fu-YinPierre, GallezotAlpuim, PedroHSU, Sam H.Y.Pietrzyk, BozenaAl-Tabbaa, AbirHu, AnmingPillai, Rajalekshmi C.Altarawneh, M.Hu, Chien-ChiehPiller, MarzioAltmann, BrigitteHu, Ching-YaoPimentel, AnaAltstadt, E.Hu, Jin-JiaPina, SandraAlvarez, Noe T.Huang, Bohr-RanPinal, RodolfoAlxneit, IvoHuang, ChaoboPinto, RicardoAmador, UlisesHuang, Chia-HuaPippan, ReinhardAman, ZacharyHuang, Chien-LinPires, E.Amane, JadaHuang, Chih-LingPires, JoãoAmano, FumiakiHuang, Chi-YenPistidda, ClaudioAmaral, Joana S.Huang, Chung-HoPistone, AlessandroAmaro, Ana M.Huang, Hsi-YaPitchumani, RangaAmeli, AmirHuang, JianweiPitsikalis, MarinosAmiri, AliHuang, JiePlacet, VincentAmsler, MaximilianHuang, KePlacidi, MarcelAn, JiaHuang, Liang-FengPlasseraud, LaurentAn, JinwooHuang, MengbingPleixats, RoserAn, ZhonglieHuang, QijinPluvinage, GuyAnasori, BabakHuang, Song-JengPoggio, ClaudioAnderson, NeilHuang, Wen-ChangPohl, MichaelAnderson, Wayne A.Hug, EricPoilane, ChristopheAndersson, Gunther G.Hummer, DanielPoisson, Jean-LouisAndo, YasunobuHumphreys, PaulPokharel, PashupatiAndrejs, LukasHunt, John F.Polimeni, AntonellaAnnamdas, VenuHuo, DehongPompe, TiloAntou, G.Hupa, LeenaPoon, S.J.Antunes, MarceloHur, DohaengPoon, Ting-ChungAoki, Ken’ichiHurst, RogerPopok, VladimirAramesh, MortezaHussain, BabarPopov, InnaAraujo, PedroHussain, N. SoorajPopp, AlexanderArbelaiz, AitorHusson, JérômePorco, FrancescoArbiol, JordiHuter, PatrikPosadas, P.Archer, RichardHutmacher, Dietmar W.Potgieter, HermanArcos, DanielHuysmans, M.-C.D.Pouhet, RaphaelleArdu, StefanoHwang, C.L.Poumellec, B.Argyropoulos, ChristosHwang, Chung-JuPoursaee, AmirAriga, KatsuhikoHwang, Nathaniel S.Poussard, LoïcArinzeh, Treena L.Hwang, SeongpilPozo-Antonio, J.S.Arnusch, ChristopherHwang, Shun-FaPrabhu, RajkumarArrua, DarioHwang, TaejinPrasetiadi, A.E.Arslan, ZikriIacoviello, FrancescoPrato, AndreaAruna, PrakashIandolo, DonataPrévot, VanessaArvapally, Ravi KumarIatrou, HermisPrice, TonyArzi, BoazIbáñez Mendizábal, RaquelPrimig, SophieAsgari, H.Iborra, EnriqueProserpio, DavideAshiuchi, MakotoIdapalapati, SridharProsperi, DavideAshkenazy, YinonIgari, T.Provine, J.Assender, HazelIglesias Castrillejo, OlaiaPsarobas, IoannisAsteris, Panagiotis G.Ihalainen, PetriPu, HaihuiAston, EricIjima, HiroyukiPudasaini, Pushpa RajAtanasov, MihailIkuma, KaoruPuertas, FranciscaAtencia, JesúsIliopoulos, KonstantinosPuertas, IgnacioAtkinson, AlanIlliberi, AndreaPuga, H.Atsushi, WakamiyaImai, HiroakiPuglia, DeboraAttik, NinaImai, MotoharuPuiggalí, J.Aubert, ThierryInazumi, ShinyaPuiggalí, JordiAuersch, LutzIncarnato, LoredanaPujadas, PabloAugustynski, JanInfante, VirgíniaPujo, Mariacinta C.Awual, M. RabiulIngham, JasonPuli, V.S.Aydemir, UmutInomata, KatsuhiroPullman, David P.Aydin, AlperInoue, YokuPumera, MartinAymard, LucIrie, MasaoPungente, MichaelAyuso, JesúsIrvine, Scott A.Pyatenko, AlexanderAzushima, AkiraIsa, LucioPylypenko, SvitlanaBaaj, HassanIshitani, YoshihiroPyo, SukhoonBabaei, HasanIslam, Mohammad SaifulPyttel, BritaBacigalupo, AndreaIsland, J.O.Qin, Chao-ZhongBackov, R.Isobe, KoichiQiu, LongzhenBadea, IldikoIto, AkihikoQu, BingBadini, ClaudioIto, MakioQuesada, AdriánBaeg, Kang-JunIto, TaichiQuignard, FrançoiseBaeza, MireiaIvana, SavicQuinn, JohnBagby, MichaelIvask, AngelaQuiñones-Mateu, M.E.Baghdasaryan, TigranIvers-Tiffée, EllenQuintero, FélixBaglio, VincenzoIvkov, RobertRaabe, DierkBagnoli, PaolaIwamoto, TakeshiRademacher, PeterBahk, Je-HyeongIwasaki, TomohiroRaftopoulos, KonstantinosBai, JinboIwata, TakanoriRagab, K.A.Bain, Colin D.Izadi, HosseinRahaman, Md. SaifurBaino, FrancescoIzumi, AtsushiRaheem, Adeeba AbdulBaitinger, MichaelJacobsen, StefanRahman, Akm SamsurBaji, AvinashJadwicienczak, WojciechRakhmonov, JovidBajorek, AnnaJaegle, Eric A.Ramadoss, A.Bajwa, Dilpreet S.Jagerovic, NadineRamazani, AliBajwa, RizwanJahan, Muhammad P.Ramon, EloiBäker, MartinJajam, KailashRana, DipakBakhshi, HadiJakes, Joseph E.Ranganathan, NaliniBakir, AdilJakka, Suresh KumarRao, BalajiBakry, RaniaJakymiw, Andrew G.Rao, HarishBalagna, CristinaJamshidi, AliRao, Kamineni P.Balandin, Alexander A.Janda, RalfRaos, GuidoBalasubramanian, KannanJang, ChangheuiRaphael, PiloBalázsi, CsabaJang, Jason Shian-ChingRaucci, Maria GraziaBaldomir, DanielJang, JinRavaioli, StefanoBalogh, AttilaJang, Seung SoonRavasio, ChiaraBaptista, PedroJanka, OliverRavelli, DavideBaranger, EmmanuelJankowski, Nicholas R.Ravelo, BlaiseBaranov, OlegJarvis, KarynRayner, MarilynBarbero-Barrera, M.M.Jassby, DavidReaney, IanBarbu, MagdaJaunich, MtthiasRebollar, EstherBarker, SusanJavadi, AliReddy, M.V.Barragán, V. MaríaJayakumar, RangasamyReddy, N.S.Barranco, AngelJayant, Rahul DevRegev, OrenBarretta, RaffaeleJayaprakash, M.Reguera, JavierBarrios, LleonardJayasinghe, SuwanReichelt, SentaBarron, Andrew R.Jean-Luc, MenetReif, JuergenBarroso, TelmaJebahi, MohamedReimanis, IvarBarroso-Bogeat, AdriánJefferies, Steven R.Remediakis, IoannisBarry, MatthewJeffs, SpencerRen, HaoBartocci, PietroJelovica, JasminRen, HuilongBarton, Larry L.Jenkins, Michael G.Ren, QiangBarton, NickJenner, Henk A.Rey, Alejandro D.Barua, BipulJensen, Ole MejlhedeRezaee, RezaBarucca, GianniJeon, HyeongtagRezvantalab, HosseinBassani, MarcoJeon, Ju-WonRheinheimer, WolfgangBassani, PaolaJeong, Doo SeokRibakov, YuriBatchelor, WarrenJeong, Han MoRibeiro, BettencourtBaumeister, JoachimJeong, Soon-JongRiccardo, ZoiaBayer, Ilker S.Jesus, Vazquez ValeoRichter, HannesBazaka, KaterynaJhon, Young MinRidi, FrancescaBeake, BenJi, GangRigamonti, LucaBear, JosephJi, VincentRighi, Maria CleliaBeard, James D.Ji, WeiRinaldi, CarlosBeazi-Katsioti, MargaritaJian, ChenRinaldi-Montes, NataliaBeck, StephanieJiang, Cho-PeiRiu, Doh-HyungBeck, TilmannJiang, JunhuaRivard, PatriceBeckford, Floyd A.Jiang, LeoRiveiro Rodriguez, A.Beckingham, Lauren E.Jiang, XiaoningRivera, PedroBedia, JorgeJiang, XuchuanRizzano, GianvittorioBedon, ChiaraJiang, ZhengyiRizzarelli, PaolaBegines, BelenJin, FeiRizzi, Gian AndreaBehnood, AliJin, PengRo, RuyenBehrens, Bernd-ArnoJin, TonyRoberto, CrespiBellantone, VincenzoJin, ZhaoRocha, CristinaBellet, MichelJitianu, MihaelaRochford, LukeBelzunce, Francisco J.Jitsangiam, PeerapongRochoux, M.C.Bemer, PascaleJiu, J.Rodrigue, DenisBen, GoichiJivkov, Andrey P.Rodriguez, BrianBen Haha, MohsenJohnson, Scooter D.Rodriguez Ripoll, ManelBenali, SamiraJohnsson, MatsRodríguez-Alloza, A.M.Bencardino, FrancescoJohnston, Karen E.Rodríguez-Castellón, E.Bendeck, Michelle P.Jomaas, GrundeRodriguez-Ubinas, E.Benedetti, I.Jones, J. CliffRoehling, Tien TranBenelli, GiovanniJones, Jacob L.Roger, LeblancBenito, N.Jones, Julian R.Roggendorf, HansBerenjian, AydinJones, RhysRogier, HendrikBeretta, StefanoJonkers, HenkRogl, PeterBergner, F.Joo, Sang-WooFrías, MoisésBernado, GabrielJood, PriyankaRomain, LucasBernardo, EnricoJoonphil, ChoiRomeo, NicolaBernardo, MariaJoos, JonasRomero, PedroBernasconi, AndreaJosé, AntonioRomero-Gómez, PabloBernd Schönbauer, BerndJothi, SathiskumarRonca, SaraBernède, Jean C.Jotshi, ChandRong, YimingBerthelot, ThomasJou, ShyankayRoper, D. KeithBertino, Massimo F.Jouini, NoureddineRoppolo, IgnazioBerto, FilippoJuan, KiwiRosa, RobertoBertrand, FrancoisJuang, Jia-YangRose, Joseph L.Bertrand, Jean-RémiJuang, Yi-JeRossi, Cesare OlivieroBhattacharya, AmritaJung, JaepilRossi, FedericoBhatwadekar, Ashay D.Jung, SeunhoRossi, René M.Bhowmick, SankhaJurado-Sánchez, BeatrizRotermund, H.H.Bi, GuijunKacena, Melissa A.Roth, JohannesBi, ZhumingKaczmarek, Anna M.Roth, Stephan V.Bichler, LukasKaewunruen, SakdiratRöttger, A.Biermann, HorstKagawa, YutakaRousakis, TheodorosBiesuz, MattiaKajihara, MasanoriRoviello, GiuseppinaBikiaris, Dimitrios N.Kakali, GlikeriaRtimi, SamiBillah, MuntasirKakio, ShojiRuark, MattBimbo, NunoKakkar, Ashok K.Ruban, AndreiBirkett, MartinKako, TetsuyaRubano, AndreaBiro, ElliotKalkur, T.S.Rubini, SilviaBiswal, Sibani LisaKamali, SaeedRubio-Marcos, FernandoBiswas, KaushikKamegawa, TakashiRuff, AdrianBiswas, ShaurjoKamei, Caramai N.Ruffino, F.Blaker, JonnyKanellopoulos, AntoniosRüggeberg, MarkusBlanc, D.Kang, Seong JunRuiz Hervías, JesúsBlanco-Valera, M.T.Kang, ThomasRuiz-Rodriguez, A.Bland, LeslieKang, XinRuiz-Rosas, RamiroBlázquez, J.S.Kang, YoungsooRulis, PaulBoanini, ElisaKao, Hsien-MingRupp, FrankBobadilla, L.F.Kao, Hui-LingRussell, Alan M.Boeffel, ChristineKao, Kuo-ShengRussell, Thomas P.Boehm, StefanKao, Po-WeRusso, Juan ManuelBokias, GeorgiosKaplan, BartekRusso, PietroBolander, JohnKaplas, TommiRusso, SalvatoreBolotov, LeonidKappera, RajeshRutner, MarcusBolouri, AmirKaraca, Haluk E.Rybakov, K.I.Bolz, SebastianKaraki, TomoakiRyou, Jae SukBolzoni, LeandroKaralekas, DimitrisRyu, Sung-SooBombelli, PaoloKarg, MatthiasRyu, Won-HeeBönisch, MatthiasKärger, JörgSa’ncheza, LuisBonse, JörnKarkri, MustaphaSablani, Shyam S.Bontempi, ElzaKarolus, MalgorzataSaccani, AndreaBoonkaew, BenjawanKarpukhina, NataliaSacco, RiccardoBordelon, AmandaKarra, SatishSacher, EdwardBoria, SimonettaKartsonakis, IoannisSada, KazukiBorkow, GadiKarygianni, LampriniSadayappan, KumarBormashenko, EdwardKashimura, KeiichiroSadeghifar, HamidrezaBornapour, MandanaKasper, F. KurtisSáez Del Bosque, Isabel F.Borodianskiy, KonstantinKasperovich, GalinaSafiuddin, M.Borrachero, M.V.Kathuria, HimanshuSaghafi, H.Borrega, MarcKatsui, HirokazuSaha, BiswajitBortot, ElianaKaunas, Roland R.Saha, GobindaBoschetti, EgistoKaur, GurbinderSaha, Sourabh K.Botti, SilvanaKaveh, EdalatiSahariah, PriyankaBoukherroub, RabahKawasaki, MegumiSahu, SulataBouquillon, SandrineKažys, Rymantas JonasSai, HitoshiBouropoulos, NikolaosKebriaei, RezaSaikia, NabajyotiBoussuge, MichelKempen, KarolienSaito, KenjiBowen, Patrick K.Kent, DamonSaito, NoritakaBradford, PhilipKenzari, SamuelSakagami, KimihiroBraga, Ángeles SanrománKeogh, MichaelSakai, EtsuoBrailovski, VladimirKern, MatthiasSakairi, M.Braissant, O.Kessler, SylviaSakalas, PauliusBranco, FernandoKettwich, Sharon C.Sakaushi, KenBrand, Alexander S.Kevadiya, BhaveshSakellariou, GeorgiosBrand, MarjorieKevern, JohnSakhno, Oksana V.Brandell, DanielKhajotia, Sharukh S.Sakkas, KonstantinosBrandenburg, Jan GeritKhandelwal, ManojSakulich, AaronBrandt, MilanKhansur, Neamul HayetSalahub, DennisBrendel, LotharKharkar, PrathameshSalak, AndreiBrennan, OrlaithKharrat, MohamedSalamone, SalvatoreBrezesinski, G.Khatib, JamalSalasi, MobinBriffaut, MatthieuKherani, Nazir P.Saldana, ChristopherBrighenti, RobertoKhodadadi, Jay M.Salerno, AurelioBriscoe, JoeKhodaei, Zahra SharifSalminen, AnttiBriscoe, WugeKhoddam, ShahinSalud, SerranoBroer, DickKhorasani, Amir MahyarSalvia, MichelleBrögelmann, T.Khovaylo, VladimirSamavedi, SatyavrataBroring, M.Khulbe, K.C.Samba, RamonaBrostow, WitoldKhun, Nay WinSamuel, FawzyBrouwers, JosKichambare, PadmakarSamyn, FabienneBrouzoulis, JimKichambare, Padmakar D.Sánchez, MercedesBrown, IanKikuchi, DaisukeSánchez-Sánchez, M.Brüggemann, OliverKim, BruceSanders, Paul G.Brunner, Andreas J.Kim, Chang-SooSangiorgi, CesareBryan, EigenbrodtKim, Dae EunSanjuán, Miguel ÁngelBu, Xiu R.Kim, Dae SuSantamaria, MonicaBueno, MoisesKim, Do YoungSantiago, Ferrándiz BouBugarin, AlejandroKim, Dong-Joo (Daniel)Santiuste, CarlosBugnet, MatthieuKim, Duck YoungSantoro, CarloBui, Bernadette Tse SumKim, Gue-HyunSantoro, LucianoBuonocore, FrancescoKim, H.Y.Santos, AbelBurbank, MalcolmKim, Hak-YongSantos, DiogoBurrows, NathanKim, Hyun JaeSantos, Maria JacintaBusche, ChristophKim, HyungsunSantos-Carballal, DavidBuschmann, Matthias H.Kim, Hyun-JoongSantulli, CarloBustillo, AndresKim, Hyun-SukSantus, C.Butt, JavaidKim, Il-HoSanz-Herrera, Jose A.Butylina, SvetlanaKim, Jae HongSapelkin, AndreiBuysser, Klaartje DeKim, JihoonSarasini, FabrizioByeon, Jai-WonKim, Jung-GuSareh, SinaCabana, LauraKim, Keun-SooSarkar, Susanta K.Cabeza, M.Kim, Ki-BuemSasaki, TakeoCabral, JaydeeKim, Kwang-MahnSasson, YoelCabrini, MarinaKim, KyungmokSassoni, EnricoCacciato, GiuseppeKim, Myong-HoSastry, Shankar M.L.Cachoncinlle, ChristopheKim, Sang JaeSato, SusumuCadoni, EzioKim, SuminSaunders, Steven R.Caggiano, AlessandraKim, Sung YoubSaunier, SebastienCagnona, BenoîtKim, SunghyunSavija, BrankoCahill, LaurenceKim, Sung-SooSawpan, Moyeenuddin A.Cai, GaochuangKim, Tae KyuSayadi, Ali A.Cai, QiongKim, TaehwanSbarufatti, ClaudioCai, ZhongyuKim, WonhoScalese, SilviaCaillol, SylvainKim, Yang SooScalet, G.Calderón, VerónicaKim, YoongScalici, TommasoCalliari, IreneKim, Young SikScarano, DomenicaCalvaresi, MatteoKinuthia, JohnScarfato, PaolaCalzaferri, GionKinzel, EdwardSchauer, CarolineCamacho, Lucy M.Kip, DetlefSchebarchov, DmitriCamblor, Miguel A.Kipouros, Georges J.Schille, C.Caminero, M.A.Kishore, VipuilSchirp, ArneCampbell, JohnKitada, AtsushiSchlaad, HelmutCamposeo, AndreaKitajou, AyukoSchlickewei, Carsten W.N.Campus, GuglielmoKlapotke, T.M.Schmid, MarkusCancelliere, PiergiacomoKlapötke, ThomasSchmidt, JochenCandolfi, ChristopheKleebe, Hans-JoachimSchmitz, MatthiasCaniato, MarcoKlenk, ReinerSchnabel, VolkerCannon, Carolyn L.Klinge, U.Schneck, EmanuelCanteli, Alfonso F.Klöcker, HelmutSchneider, GerhardCapdevila, CarlosKlöden, BurghardtSchneider, RaphaëlCarbonnier, BenjaminKlug, Dennis D.Scholes, ColinCarette, JérômeKnowles, KevinSchöneich, MarcCarlone, PierpaoloKo, ChanghyunSchroefl, ChristofCarneiro, O.S.Ko, Jang MyounSchubert, Th.Caroni, C.Ko, Seung HwanSchütz, ChristinaCarrado, AdeleKo, Young GunSchwarz, GuntramCarriel, VíctorKobayashi, MakikoSchwendicke, FalkCarrino, LuigiKobayashi, SatoshiScialdone, OnofrioCarry, ClaudeKoduru, Janardhan ReddyScott, JasonCaruntu, GabrielKoeller, ManfredScott-Emuakpor, O.Casalino, GiuseppeKoenders, EddyScrosati, ChiaraCasettari, LucaKoga, Jun-ichiroSeetharaman, S.Castagne, SylvieKoissin, VitalySeidlits, StephanieCastaño, OscarKoizumi, HiroyasuSelleri, StefanoCastillo, JaimeKoizumi, ToshioSellin, Paul J.Castillo-Martinez, E.Kojima, KazutoshiSemagina, NataliaCastro, AndreKolasinski, Kurt W.Semaltianos, N.G.Castro-Gomes, JoãoKolehmainen, ErkkiSemino, Carlos E.Catala, LaureKolesnichenko, Vladimir L.Sen, SanghamitraCatauro, MichelinaKomasa, SatoshiSenyuk, BohdanCavalline, TaraKomnitsas, K.Seo, Hyo JinCavallini, MassimilianoKonda Gokuldoss, P.Seo, Young-SooCavallotti, PietroKondoh, KatsuyoshiSeong Sik, HwangCavicchi, KevinKonegger, ThomasSerena, SaraCelano, UmbertoKontic, RomanSerra, AngelsCelle, CarolineKonwar, Lakhya JyotiSerra, RogerCelli, MicheleKonya, ZoltanSerralha Dos Anjos, O.M.Cesano, FedericoKopsaftopoulos, FotisSerrano, SusanaCeyssens, FrederikKordesch, Martin E.Sesana, RaffaellaChaboussant, GregoryKoren, KlausSevastyanova, OlenaChailleux, EmmanuelKorner, CarolinSevilla, PabloChaim, RahmanKorposh, SerhiySevostianov, IgorChalioris, Constantin Ε.Koschek, KatharinaSeyedin, ShayanChamanfar, AhmadKostoglou, MargaritisShabadi, RajashekharChampagne, Victor K.Kotek, R.Shacham-Diamand, YossiChan, Candace K.Kothapalli, C.R.Shafei, BehrouzChan, JerryKotobuki, MasashiShafiei, MahnazChan, Yang-HsiangKotsakis, Georgios A.Shah, PriyankChandrappan, J.Koukouzas, NikolaosShahinpoor, MohsenChandrasekaran, N.Kourkoumelis, NikolaosShalom, MennyChang, Chih-YuKovalevsky, Andrei V.Shang, LiChang, Chi-JungKowalska, EwaShanmugharaj, A.M.Chang, Huan-ChengKozliak, Evguenii I.Shao, HuaiyuChang, KunKranz, StefanSharp, John M.Chang, S.Y.Krasniewska, KarolinaShaygan, MehrdadChang, Sheng-PoKreouzis, TheoShehata, MedhatChang, Tso-Fu MarkKretinin, Andrey V.Shen, ShoucangChang, Wei-JenKrifa, MouradShen, Yung-KangChang, Young-WookKrim, J.Shercliff, HughChao, JesusKrishna, LakshmiShi, SuanChao, Sao-JengKrishnamurthy, AjayShi, YunhuaCharalambides, Panos G.Kroll, Daniel M.Shih, Ming-ChengChase, George G.Krstulovic Opara, LovreShih, Shao-JuChau, Joseph Lik HangKrüger, JensShih, Wen-PinChauhan, MoniKrumeich, FrankShimamura, YoshinobuChaunsali, PiyushKruopienė, JolitaShimoni, OlgaChave, TonyKruppke, BenjaminShin, Boo YoungChen, BiaoKuang, HuihuiShin, D.Chen, ChunhuiKuang, WenjunShin, Eun JuChen, Chun-TaKube, C.M.Shin, KwanwooChen, HaofengKubicki, JamesShin, SwaneeChen, Jian-LianKucerik, JiriShinde, SachinChen, Jian-ZhangKuktaite, RamuneShinobu, HashimotoChen, Ko-ShaoKulagin, RomanShinoda, YutakaChen, NingKulkarni, NehaShinya, NorioChen, RobertKumar, Annamalai P.Shipilov, SergeiChen, RogerKumar, AdityaShirai, NaokiChen, Ruei-SanKumar, DeepakShirvanimoghaddam, K.Chen, RuiyongKumar, DhananjayShishido, AtsushiChen, ShuangqiangKumar, NareshShokouhi, ParisaChen, ShujianKumar, RajShrestha, Lok KumarChen, SimingKume, TetsujiShu, XiangChen, Tao-HsingKunieda, MinoruSiad, HocineChen, XiaoboKuniharu, UshijimaSiddiq, M. AmirChen, YiKunjukunju, SangeethaSierra, Miguel AngelChenal, Jean-MarcKuo, Chil-ChyuanSignoretto, MichelaCheng, AnKuo, ShiaoweiSilva, F.J.G.Cheng, Chil-HungKuo, Wen-TenSilva, M.M.Cheng, Fong-YuKuo, Y.K.Silva, Rui VascoCheng, JipingKurakevych, Oleksandr O.Silvestre-Albero, JoaquinCheng, Ko-TingKurashina, MasashiSilvestroni, LauraCheng, Kou-BingKurganov, A.A.Simka, WojciechCheng, QianKuršelis, KęstutisSimmonds, Paul JCheng, RuiKurz, DanielSimmons, TrevorCheng, ShiwangKusch, PeterSimões, SóniaCheng, XingguoKustov, SergeySimone, SprioCheng, Yang-TseKutuvantavida, YasarSims, IanChen-Yang, Yui-WheiKuwahara, YasutakaSing, Swee LeongCherevan, AlexeyKuzmikova, LenkaSingamneni, SaratChesneau, XavierKwok, Kin WingSingh, AlokChettle, DavidKwon, Han-SangSingh, AshishCheung, Angus K.F.Kwon, Il-KeunSingh, GurpreetChi, Mao-ChiehKwon, Jang-YeonSingh, Preet M.Chiachio, ManuelKwon, Nam HeeSingh, RanjanChiam, S.Y.Kwon, Oh-SunSingh, RanjeetChiang, Chin-LungKwon, Tae-yubSinha Ray, SumanChiang, Long Y.Kyratsis, PanagiotisSirman, AsliChiappone, AnnalisaKyu, Kim EunSirusta, SilviaChiarello, Gian LucaKyu, TheinSisson, Richard D.Chidambareswarapattar, C.La Mantia, Francesco P.Sista, SubhashChien, Chao-hsinLabat, BeatriceSivakumar, K.Chiesa, RobertoLabzowskaya, M.E.Sjogren, GoranChiessi, EsterLagos, M.A.Skolianos, Stefanos M.Chikamatsui, AkiraLaguerre, LaurentSkoulou, Vassiliki K.Chikan, ViktorLaguna-Bercero, Miguel A.Skov, AnneChikara, ShalineeLai, Jui-YangSkrifvars, MikaelChin, W.S.Laidani, Nadhira B.Skurla, CarolynChiono, ValeriaLal, AshwinSkuza, Jonathan R.Chirea, MarianaLamberti, AndreaSlugovc, ChristianChiu, Wilson K.S.Lamberti, L.Smått, Jan-HenrikCho, DonghwanLambiase, FrancescoSmialek, James L.Cho, In SunLancelloti, IsabellaSmiatek, JensCho, Jae UngLanga, SergiuSnitka, ValentinasCho, Jae-HyungLanghans, Sigrid A.Snoeck, DidierCho, Sung-JinLantieri, ClaudioSnyder, JoshuaCho, YongJinLao, JonathanSoares, Olívia S.G.P.Chobot, VladimirLaoutid, FouadSobey, A.J.Choi, AndyLapington, JonSobolev, KonstantinChoi, Chel-JongLaptev, AlexanderSödergård, AndersChoi, Inhee CurieLarosa, ClaudioSoenen, HildeChoi, Yoon-SeokLarsson, ChristelSohn, YoungkuChoudhary, DipankarLattuada, MarcoSokolis, Dimitrios P.Chowdhury, PiyasLau, RaymondSolano, WilberthChristoforidis, K.C.Laudone, GiulianoSoleimani Dorcheh, AliChu, BenjaminLaurent, BechouSoliman, Ahmed M.Chung, Chan-MoonLaurenti, MarcoSolioz, MarcChung, Gui YungLaurin, FredericSolitro, GiovanniChung, Kwok-HungLauth, Jannika D.Soltani, AliChurch, Benjamin C.Law, D.W.Son, Ji-WonChyrvony, VladimirLaycock, BronwynSonebi, MohamedCiapetti, GabrielaLázár, IstvánSong, KenanCiavarella, MicheleLazzara, GiuseppeSong, Nam WoongCicala, GianlucaLe Breton, Jean-MarieSong, WeiminCicciu, MarcoLe Guennic, BorisSong, XuCicero, SergioLe Moigne, N.Song, Young MinCicogna, FrancescaLebeau, BenedicteSorelli, LucaCidade, M. TeresaLechler, PhlippSorgente, DonatoCipres, Miriam SolanaLeclère, PhilippeSornin, DenisClark, AlasdairLecompte, ThibautSorrentino, AndreaClarke, Thomas A.Lecomte, PhilippeSotta, PaulClaver, CarmenLedi, MenabueSoyarslan, CelalClement, MartaLee, B.Y.Speliotis, T.Climent, Miguel ÁngelLee, ChanSpena, Pasquale RussoClouston, Peggi L.Lee, Chia-hungSperanzini, EmanuelaCnudde, VeerleLee, GilSpiess, LotharCoakley, EoinLee, Hee YoungSpiesz, PrzemekCobo, AlfonsoLee, Hian KeeSpilotro, GiuseppeCoco, SilverioLee, Hung BinSpoljaric, StevenCodispoti, RosamariaLee, Hyuck MoSproules, StephenCodrington, JohnLee, In-SeopSrinivasan, MadapusiCoelho, João M.P.Lee, Jae-ShinSrivastava, AnkitCoffinier, YannickLee, Jae-SukStamatakis, MichailCohn, GilLee, Ju-HyoungStampfl, CatherineCoiffard, Laurence J.M.Lee, JunghoonStanzione, JosephColangelo, GianpieroLee, Jung-MooStathis, BousiasColegrove, PaulLee, JungwooStavrinadis, AlexandrosColeman, NicholaLee, Jun-JaeStefan, HolmstromCollini, LucaLee, JustineStefanidou, MariaCollins, MauriceLee, Kang TaekStefanou, GeorgeCollins, ScottLee, Kew-HoStephan, DietmarColom, XavierLee, Maw TienStevenson, Adam J.Condoret, J.-S.Lee, Ming-GinStevenson, TimothyConings, BertLee, Myoug-GyuStinville, J.C.Conner, Robert D.Lee, Sang JoonStock, S.R.Consoli, AntonioLee, Sea-HoonStöcker, AnettConstable, Edwin C.Lee, Seung-Woostramul, Boris B.Conti, BiceLee, Wei LiStrangwood, MartinCools, PieterLee, Wen-JauStraumal, Boris B.Coon, DennisLee, Woei MingStrehmel, BerndCopelli, SabrinaLee, Won JaeStride, John ArronCorcione, C. EspositoLee, WoongStrini, AlbertoCorcione, Carola E.Lee, Y.C.Ström, AnnaCórdoba, José ManuelLee, Yiu Yin RaymondStrozzi, MatteoCoronas, JoaquínLefort, RonanStrunk, JenniferCorpas-Iglesias, FranciscoLehenkari, PetriStuart, Marc C.A.Cortese, BarbaraLehmhus, DirkStumpel, JelleCosma, PinalysaLehmkühler, FelixSu, CarrieCosta, HugoLeidermark, DanielSu, ChunmingCosta, Mário M.G.Lekka, ChristinaSu, Yu-MinCostentin, GuylèneLemu, Hirpa G.Su, ZhouchengCoutu, RonaldLeng, YangSuarez, Maria JesusCozzoli, P. DavideLeng, ZhenSuay Antón, Julio JoséCrabtree, Robert H.León, GerardoSubramaniam, PrasadCran, Marlene J.Leonelli, CristinaSuckeveriene, RanCrane, Cortney B.Leporatti, StefanoSugahara, T.Creamer, Anne EliseLeskelä, MarkkuSugavanam, SrikanthCristelo, Nuno MiguelLeung, Christopher K.YSugawa, KosukeCristiani, PierangelaLevesque, MartinSuib, Steven LCristino, VitoLevi-Polyachenko, NicoleSuk, Ji WonCroccolo, DarioLevitas, Valery I.Sukhishvili, SvetlanaCrossland, W.A.Lewis, DavidSun, HongqiCrupi, VincenzoLeyva-Pérez, AntonioSun, KaiCui, FangsenLi, HuiSun, Kien WenCui, HaitaoLi, HuijunSun, TaoCulliton, DavidLi, J.F.Sun, WenpingD’Lima, Darryl D.Li, JiantongSun, ZhidanD’Alessandro, AntonellaLi, JiashenSun, ZiqiD’Alessandro, FrancescoLi, JichaoSundaram, Kalpathy B.Dalton, PaulLi, JieSundaram, MuraliDamodaran, KrishnanLi, JinghaoSundaram, PaulDa’na, EnshirahLi, Kai ChunSung Ho, YangDang, CuongLi, L.H.Susan, Donald F.Dante, Roberto C.Li, Long-YuanSuzuki, SeiichiDaoud, Walid A.Li, LuhuaSwolfs, YentlDarling, SethLI, Robert Kwok-YiuSydlik, Stefanie A.Darmanin, ThierryLi, ShaofanSydoruk, OleksiyDas, AmitLi, WeiyangSzwajka, KrzysztofDa Silva, Hugo M.R. DiasLi, X.P.Tabaković, AmirDassios, Konstantinos G.Li, XiangyuTabata, YasuhikoDatcheva, MariaLi, XiaodongTaboada, PabloDauscher, AnneLi, XiaojunTabuchi, MasaakiDauskardt, Reinhold H.Li, Yan (Vivian)Tafraoui, AhmedDavis, ChelseaLi, ZhanjunTafu, MasamotoDavis, Daniel C.Lian, J.Tagawa, HiroshiDavis, KristopherLianos, PanagiotisTaheri, AbbasDavy, JohnLiao, Chih-HsiangTahriri, MohammadrezaDawes, JudithLiao, K.-C.Takabatake, ToshiroDawson, PhilLiao, SusanTakahashi, KeiDe Aza, Piedad N.Liao, YouguiTakamizawa, ToshikiDe La Fuente, Germán F.Liao, Yunn-ShiuanTakano, ToshiyukiDe La Torre, Angeles G.Liau, Wen-BinTakata, TsuyoshiDe Llano, Dolores G.Lichtenegger, HelgaTakeguchi, TatsuyaDe Lima, Mauricio M., Jr.Lichti, Keith A.Takeshita, SatoruDe Los Santos, Desiree M.Liguori, BarbaraTakeuchi, EstherDe Souza, GraceLikodimos, VlassiosTakeya, SatoshiDe Matteis, GianfrancoLim, Chang-sungTakoudis, Christos G.De Marchi, LucaLim, Jae-HanTalaat, AhmedDe Marco, LuisaLim, Yee YanTallarico, MarcoDe Martino, LauraLimberger, HansTam, Chat-TimDe Oliveira Rocha, A.M.Lin, Chi-ChangTamborrino, M.De Peppo, Giuseppe M.Lin, Chih-MingTamburrano, P.De Rességuier, ThibautLin, Ching HsuanTan, Chung-SungDe Sio, LucianoLin, Feng-HueiTan, Lay PohDe Teresa, José MariaLin, Gong-RuTan, Noel Peter BengzonDe Villoria, Roberto G.Lin, I-NanTan, Say HwaDe Vries, RenkoLin, Jong-LiangTan, TingDecker, SabineLin, Pai-ChenTanaka, AtsuhiroDeganello, FrancescaLin, SidneyTanaka, ManabuDegardin, AnnickLin, Su-JienTanaka, T.Del Valle, Luis J.Lin, Tai-YuanTanaka, YoshinobuDelacy, BrendanLin, Yen-HuiTanemura, KiyoshiDelannay, FrancisLin, YouxiTang, FujianDelaunois, FabienneLin, Yuan-HuaTang, P.Delgado, Juan LuisLin, YuankunTanii, TakashiDelgado, MiguelLing, BoTanksale, AkshatDelgado, MónicaLing, Maurice HtTarfaoui, MostaphaDelichatsios, MichaelLink, GuidoTaruta, SeiichiDelongchamp, DeanLinko, VeikkoTastani, Souzana P.Delpero, TommasoLinsley, ChaseTatemoto, YujiDelpouve, NicolasLionetti, VincenzoTaubert, AndreasDemis, SotirisLipinski, P.Tavangarian, FariborzDenizot, B.Lipomi, Darren J.Tavares, Carlos J.Deravi, Leila F.Liss, Klaus-DieterTavares, João ManuelD’Este, MatteoLitina, ChrysoulaTavío, María Del MarDesvergne, Jean PierreLiu, Chi-HsienTaylor, AdamDeterre, RémiLiu, Hong YuanTeisala, HannuDeville, SylvainLiu, JinyunTena-Zaera, RamonDevlin, J. PaulLiu, K.Teng, HsishengDezecot, SebastienLiu, Lin ShuTengstrand, OlofDhakshinamoorthy, A.Liu, MinTerada, KenjiroDhanasekar, ManickaLiu, RuiTerAvest, MichaelaDhir, AmanLiu, SongTerry, IanDi Bartolomeo, AntonioLiu, Sung-PoTestoni, NicolaDi Bella, GuidoLiu, YanbiaoTexier, DamienDi Luzio, GiovanniLiu, YeTexter, JohnDi Maggio, RosaLiu, Ying-LingTextor, TorstenDi Maio, ErnestoLiu, ZhenTezel, F. HandanDI Martino, PieraLiu, ZhichaoTezvergil-Mutluay, ArzuDi Pasqua, AnthonyLloret, AntonioThackray, RichardDias-da-Costa, DanielLo, Fang-YuhThadhani, NareshDickens, TarikLo Re, GiadaThakur, Vijay KumarDietrich, DagmarLöbmann, PeerThemistou, EfrosyniDíez Barra, EnriqueLocock, Katherine E.S.Thiéry, VincentDillon, FrankLodeiro, PabloThomas, Robert J.Dimakis, NicholasLohfeld, StefanThomas, SabuDimitrio, ManolakosLoizidou, ErikaThomas, SamuelDing, Shinn-JyhLong, Gary J.Thomas-Seale, LaurenDing, XinLongoni, GianlucaThompson, Richard L.Dinia, AzizLopes, CatarinaThorat, SanjayDionisi, DavideLópez De Lacalle Marcaide, L.N.Thorimbert, SergeDisfani, Mahdi M.López-Garzón, F.J.Thygesen, AndersDismukes, G. CharlesLópez-Sabirón, Ana MaríaTian, Gui YunDo, Sun HeeLorenz, MichaelTian, YuanDo Van, PhucLorenzo, MiguelTian, ZhenhuaDoert, ThomasLorusso, MassimoTibbetts, Katharine M.Domingo, RosarioLotti, NadiaTidu, AlbertDong, GuangnengLoura, Luís M.S.Timelli, GiulioDong, H.B.Louzguine, DmitriTiraferri, AlbertoDong, YalinLoveridge, MelanieTiryakioglu, MuratDorfmann, A.Lower, BrianTiwari, AshutoshDoroudiani, SaeedLu, ChengTobler, Dominique J.Dorri Moghadam, A.Lu, Fa ChuangToca-Herrera, José LuisDorris, Kenneth L.Lu, John C.Todoroki, AkiraDowns, John AxelLu, XiaoyunToita, RikiDoyle, Barry J.Luan, XianghongTolnai, D.Dragoe, NitaLubarda, V.A.Tomasi, RobertoDrelich, JaroslawLucchetta, DanieleTomaz, Cândida TeixeiraDrummond, CarlosLúcia, SantosTomita, YasuoDu, HongjianLuciani, PaolaTomohiro, YokozekiDu, XushengLuck, RudyTomoyoshi, NishiumuraDuarte, Ana Rita C.Luhrs, ClaudiaTondi, GianlucaDuba, KurabachewLuna, YolandaToo, Heng-PhonDubey, AshishLuo, He-KuanToonen, Ryan C.Ducobu, FrançoisLuo, QuanshunToraldo, EmanueleDuczek, SaschaLuong, Dzung DinhToribio, J.Duffy, AlistairLupascu, Doru C.Torimoto, TsukasaDujardin, ChristopheLuukkonen, TeroTornabene, FrancescoDunne, NicholasFregoso, Benjamin M.Torrens-Serra, JoanDunstan, ColinMa, HongyanTotolin, VladimirDuong, Hai M.Ma, HongyangToubal, LotfiDurão, Luís Miguel P.Ma, JeffToupance, ThierryDurejko, TomaszMa, X.Traini, ToninoDurual, StéphaneMa, YingTravesset, AlexDutta, DebarunMaalouf, AzarTrefalt, GregorDutta, P.K.Maas, MichaelTriantafillou, ThanasisDuvigneau, JoostMachado, Christiano B.Tricoli, AntonioDyer, TomMachado, JorgeTroadec, CedricDzenis, Yuris A.Machado, RaulTroiani, EnricoDziedzic, DominikMaçôas, ErmelindaTronci, GiuseppeEberl, HermannMacquarrie, DuncanTrtik, PavelEgawa, YuyaMacyk, WojciechTrueba, M.Eguiazábal, José I.Maderuelo-Sanz, RubénTruong, VinhEhrlich, HremannMaeda, HirotakaTruster, TimothyEigler, SiegfriedMaekawa, KoichiTsai, Cheng-FangEik, MarikaMaeno, TomoyoshiTsai, De-HaoEinarsrud, Mari-AnnMaffezzoli, AlfonsoTsao, Chia-WenEklund, Sven E.Maienschein, JonTsao, Lung-ChuanElbadawy, HosseinMaier, GerhardTsay, Hien-YieElder, SteveMaiwa, HiroshiTschierlei, StefanieElena García-Gareta, E.Makri, SofiaTsetsekou, A.Elgorban, AbdelahMalaiškienė, JurgitaTsiakaras, PanagiotisEliche-Quesada, DoloresMalandrino, G.Tsimas, StamatisElisabetta, GariboldiMalara, GiovanniTsioulou, OuraniaEliyan, FaysalMalavasi, GianlucaTsitsilianis, ConstantinosEllahi, RahmatMalda, JosTsivintzelis, IoannisEl-Safty, Sherif A.Malfatti, LucaTsoi, JamesElSawy, Ahmed H.Malinauskas, MangirdasTsompanakis, YiannisElseifi, MostafaMalkoc, VeysiTsuji, TakeshiElsener, BernhardMalucelli, GiulioTsukada, ShinyaEmin, BayraktarMamidala, RamuluTsukamoto, SusumuEmmerling, FranziskaMan, DulaTsuruoka, TakaakiEndris, VolkerManan, SamjidTsutaoka, TakanoriEngler, OlafMander, JohnTunega, DanielEnglund, Karl R.Manfredi, NorbertoTyler, LudmilaEngstrom, DanielManna, CesarUbertini, FilippoEnnas, GuidoManna, Rudra SekharUccelletti, DanielaEnomoto, M.Mannino, SaverioUchikoba, FumioEom, KilhoMansfield, ElisabethUddin, AshrafEpifani, MauroMante, Francis K.Uddin, MohammadEramo, GiacomoManthina, VenkataUeshima, N.Erarslan, NazifeManzi, StefaniaUgues, DanieleErb, UweManzoli, MaelaUkrainczyk, NevenErchiqui, FouadMao, ChuanbinUllbrand, JenniferErdem, EmreMao, YuanbingUlven, ChadErick, OgamMarcy, Zenobi-WongUmehara, NoritsuguEriten, MelihMarder, Michael P.Umeton, CesareErné, Ben H.Marder, Todd B.Umezu, IkurouEsaka, H.Marinel, SylvainUppstu, PeterEscobar, IsabelMarinoni, NicolettaUr, S.C.Esin, Vladimir A.Marketos, GeorgeUrban, PetrEspinach, Francisco JavierMarkopoulos, Angelos P.Urbanic, R.J.Estefania Rojas-Hernandez, R.Marrocchi, AssuntaUrbassek, Herbert M.Estournes, ClaudeMarszalek, MartaUrieta, JoséEugenio, FazioMartin, RachelUrness, Adam C.Evaggelia, PapiaMartin, T.J.Ushiki, IkuoEvans, Philip D.Martin-Alfonso, J.E.Uskokovic, VukEwels, ChrisMartinelli, EnzoUsui, KenjiEymery, J.Martowicz, AdamUyama, HiroshiFaber, HendrikMarzani, AlessandroVaiano, VincenzoFabregat, AzaelMasahiko, MatsubaraValencia, SusanaFabris, LauraMaschmann, MatthewValentini, LucaFaccio, GretaMascia, LenoValenza, AntoninoFahami, AbbasMasi, GiuliaValov, IliaFairclough, SimonMasoumi, HosseinVan Citters, Douglas W.Falchetto, Augusto C.Massaglia, GiuliaVan Der Mei, Henny C.Falciglia, Pietro P.Mateos-Timoneda, M.A.Van Renterghem, WouterFaleschini, FloraMathaudhu, SuveenVan Den Heede, PhilipFalmbigl, MatthiasMatsubara, HirokiVan Humbeeck, JanFalson, PierreMatsui, IsaoVan Langenhove, LievaFantozzi, GilbertMatsukawa, MamiVan Der Vurst, FaridFaraz, AliMatsumoto, KozoVandewalle, LucieFarhidzadeh, AlirezaMatsushita, TaishiVandi, LuigiFarkoosh, A.R.Maurotto, AgostinoVane, Leland M.Faruqi, Mohamed A.May, Steven J.VanEpps, J. ScottFaruqi, Mohammed A.Mayer, ThomasVangsness, C. ThomasFaulkner, R.G.Mayeur, Jason R.Vanli, O. ArdaFaupel, FranzMazza, FabioVaquette, CedryckFauzi, IlianaMazzotta, FrancescoVaresano, AlessioFawzy, Amr SherifMcCrory, JohnVargas, MarcosFear, EliseMcDonald, ArmandoVarlamov, SergeyFeczkó, TivadarMcHugh, PeterVartiainen, JariFei, BinMcKee, Marc D.Vasdravellis, GeorgeFeih, StefanieMcnelley, Terry R.Vasile, CorneliaFeldmann, ClausMeausoone, Pierre-JeanVasilopoulou, MariaFelicelli, Sergio D.Meda, A.Vastola, GuglielmoFeliu, SebastianMedri, ValentinaVatamanu, JenelFelpin, Francois-XavierMedvedovski, EugeneVázquez-Córdova, S.A.Feng, YingMeerman, HansVehmeyer, JostFenn, MichaelMeingast, ChristophVeiga, RosárioFenning, DavidMeka, S.R.Vekinis, GeorgeFergani, OmarMelling, DanielVelasco Ortega, EugenioFermani, SimonaMelro, António RuiVelazquez, Omaida C.Fermor, HazelMemarzadeh, KavehVelikov, Krassimir P.Fernandes, IsabelMendis, ChaminiVeludo, Joao PauloFernandes, Jose ValdemarMeneghetti, GiovanniVemuri, RamaFernandes, P.R.Menendez, RosaVenditti, IoleFernandes, SuseteMenezes, PradeepVendrell Villafruela, X.Fernández, LucíaMeng, F.Q.Verduzco, RafaelFernandez-Garcia, MartaMeng, LeVergani, LauraFerone, ClaudioMeng, LijianVermang, BartFerrándiz-Mas, VerónicaMeng, QingshiVernardou, D.Ferrara, LiberatoMeng, XinVickery, KarenFerraro, PietroMenger, FredricVidal, HilarioFerrier, E.Mengucci, PaoloVieira, Castorina SilvaFerro, GabrielMenna, EnzoViggiani, GioacchinoFerron, RaissaMenzel, RobertVignali, ValeriaFettkenhauer, ChristianMenzemer, CraigVignoles, GérardFey, TobiasMeraghni, FodilVila, AnnaFiedler, BodoMergheim, JuliaVilardell, MartaFierro, VanessaMeroni, DanielaVilé, GianvitoFigueira, RitaMeshi, LouisaVilela, AliceFiore, VincenzoMessina, FrancescoVilémová, MonikaFirrincieli, A.Messori, LuigiVilla, AlbertoFischer, Franz DieterMetaxa, Zoi S.Villa, ElenaFischer, Hartmut R.Metje, NicoleVillares, AnaFischer, HolgerMeulenberg, Robert W.Villaseñor Camacho, JoséFletcher, AshleighMeunier, MichelVincent, DidierFlorczyk, Stephen J.Michalsky, RonaldVisaveliya, NikunjkumarFlury, SimonMickelson, Alan R.Visconti, Richard P.Follain, NadègeMickovski, Slobodan B.Visintin, PhillipFong, EileenMikkelsen, AndersVisscher, PieterFonseca, JoanaMilanesio, MarcoVitale, AlessandraFont, JosepMilioto, StefanaVohra, Yogesh K.Foo, Guo ShiouMilitzer, MatthiasVoisey, Katy T.Foot, PeterMiller, Jordan S.Volkmer, DirkForcellese, ArchimedeMills, ChristopherVrana, Nihal EnginForest, SamuelMills, DavidVynnycky, MichaelFormia, AlessandraMimachi, H.Wada, HiroyukiFortenberry, RyanMin, JunyingWaddell, NeilFossum, EricMin, YounjinWagner, StefanFoster, Craig D.Minamikawa, HajimeWaite, Matthew M.Foti, DoraMinelli, FaustoWakisaka, MinatoFouladirad, MitraMines, R.A.W.Walcarius, AlainFox, KateMingareev, IlyaWalia, SumeetFrage, NachumMingler, BernhardWalock, MichaelFraile-Garcia, EstebanMino, LorenzoWalsh, F.C.França, RodrigoMiquelard-Garnier, G.Walsh, William R.Francesco, NegroMiraftab, MohsenWalter, XavierFranco, José M.Miranda, G.Walther, FrankFranklin, EvanMiranda, Roberto N.Wan, FengFranssila, SamiMiras, Haralampos N.Wan, Kai TaiFrazier, CharlesMiri, Amir K.Wang, Chih-FengFrediani, PieroMirihanage, W.U.Wang, FeiFrench, DavidMiriyev, AslanWang, Hai-QiaoFressengeas, C.Mirmohammadi, H.Wang, HaoFriák, MartinMiron, Richard J.Wang, HongliangFricke, KatjaMirshekar-Syahkal, D.Wang, JiFriedt, Jean-MichelMirzahosseini, M.Wang, JianyunFrostevarg, JanMishra, Yogendra K.Wang, JunFu, ChenMisra, R.D.K.Wang, LiangFu, LiMitchell, SharonWang, LiliangFu, LiansheMitraki, AnnaWang, NingFu, YongzhuMitrašinović, A.M.Wang, QinyiFuentes-Cabrera, MiguelMitsuhiro, TerakawaWang, ShaobinFuhrmann-Lieker, T.Miura, SeijiWang, ShuFujii, GarudaMiyaji, HirofumiWang, Tzu-WeiFujii, SatoshiMiyazaki, ToshikiWang, XiaomingFujii, SyujiMizoshiri, MizueWang, Xiao-YongFujii, YoshihisaMizuuchi, KiyoshiWang, XiebinFukuda, TakashiMo, KunWang, Y.M.Fukumura, TomoteruMo, WeiweiWang, YanFukushima, ManabuMobilio, NicolaWang, YapeiFukuta, SatoshiMoeini Sedeh, MahmoudWang, YixiangFunehag, JohanMogyorosi, KarolyWang, YuanFurukawa, HiroyasuMohamed, Abdul R.Wang, Yun-CheGabay, AleksandrMohamed, MostafaWang, YuxinGakhar, RuchiMohanta, AntaryamiWard, L.P.Galan-Vidal, Carlos A.Mokaya, RobertWard, LiamGalbusera, FabioMokhtari, OmidWarner, MarkGalca, A.C.Moll, JochenWarren, Nicholas J.Gales, JohnMolnar, GaborWäsche, RolfGalindo-Nava, E.I.Momma, KoichiWaske, AnjaGallego, JuanMonchau, FrancineWatanabe, MotonoriGallego, SergiMononen, MikaWatts, DavidGambardella, AlessandroMonot-Laffez, IsabelleWeber, Franz E.Gambarova, Pietro G.Monroe, Mary Beth B.Weber, KlausGamboa, ErwinMontanaro, LucioWegrzyn, MarcinGan, YixiangMontes, CarlosWehner, MartinGanne-Chédeville, C.Monti, MarcoWei, Da-HuaGao, MinMonticelli, C.Wei, GangGao, PengMoon, Doo KyungWeidner, AnjaGao, ZhiqiangMoon, JuhyukWeinberg, Annelie M.Garboczi, Edward J.Morales-Cid, GabrielWeis, StephenGarbovskiy, YuriyMorales-Palma, D.Weise, JörgGarcia, RosarioMorandeau, AntoineWeiss, PierreGarcía, JuanMoreno, PabloWeiß, SabineGarcía Calvo, José LuisMoreno-Atanasio, R.Weissenrieder, J.García Navarro, JustoMorera, Fabiola VilasecaWeng, QunhongGarcía-Garrido, Sergio E.Morgan, AlexanderWestafer, Ryan S.Garcia-Mateo, C.Mori, KohsukeWhite, BlanaidGarcia-Moreno, F.Moridi, AtiehWhite, Mary AnneGarcia-Segura, SergiMoriga, ToshihiroWhiting, MarkGardner, DianeMorita, ShigekiWhittaker, Mark T.Garg, AkhilMorkoc, HadisWhittaker, Michael R.Garner, Angelia D.Moroz, AdamWibowo, DavidGarrett, David J.Morreale, MarcoWicht, MichaelGaynor, WhitneyMorris, AmandaWidjonarko, N. EdwinGazquez, ManuelMorris, MichaelWierer, Jonathan J.Gebeshuber, Ille C.Morrisey, JerryWierichs, Richard J.Gečys, PauliusMortazavi, BohayraWiersma, Diederik S.Gélébart, LionelMortello, MichelangeloWilkinson, TimGeletii, Yurii V.Moseke, ClausWille, KayGendre, MathieuMoser, Robert D.Willerth, Stephanie M.Gentile, PiergiorgioMosey, StephenWilliams, VanceGevorgian, SpartakMostafa, AhmadWillis, David A.Ghadbeigi, HassanMotta, AntonellaWilson, Benjamin P.Ghaderi, AlirezaMouahid, AdilWilson, Lee D.Gharaibeh, BurhanMoure, AlbertoWimbush, StuartGhetmiri, Seyed AmirMower, Todd M.Windmann, MatthiasGhiassi, BahmanMozharivskyi, YurijWithayachumnankul, W.Ghidelli, MatteoMücklich, FrankWitkowski, ArturGhiotti, AndreaMuench, FalkWits, Wessel W.Ghomashchi, RezaMuguruma, HitoshiWittreck, HubertGhorbani Moghaddam, M.Muley, PranjaliWlodarczyk, Krystian L.Ghoshal, SushantaMüller-Buschbaum, P.Wolf, BettinaGiannakos, KonstantinosMungan, Carl E.Woll, ChristofGiaouris, EfstathiosMünzenrieder, Niko S.Wolny, JuliuszGiardini, C.Murai, KazukiWondraczek, LotharGiersig, MichaelMurase, NorioWong, Ching-PingGies, HermannMurcia-López, S.Wong, Dana E.Gigante, Giovanni E.Murillo, John S. RivasWong, EugeneGil, A.Muromachi, SanehiroWong, Ka-LeungGil, Francisco JavierMurphy, Ciara M.Wong, Lai MunGilardi, ElisaMuseur, L.Wong, S. SimonGinn, JamesMuttil, PavanWoodman, TimGiorgio, IvanMyers, OliverWright, CatherineGiourntas, LamprosMysliwiec, TamiWu, Chung-ChihGiraud, StephaneNadal-Gisbert, Antonio V.Wu, Ho-ShingGirot, Franck AndréNadzhafova, OksanaWu, Hsuan-ChungGirousi, StellaNagumothu, Kishore B.Wu, Hwai-ChungGizdavic-Nikolaidis, M.R.Naik, Kiran S.Wu, JinsongGjokas, MargaritisNair, Arun K.Wu, KevinGlasser, WolfgangNair, Jijeesh R.Wu, Ming-ChungGloria, AntonioNaito, YoshihitoWu, Shih-ChingGockenbach, ErnstNakajima, HideoWu, Shu-PaoGoel, SauravNakamura, YoshinobuWu, T.Goeuriot, DominiqueNakashima, Philip N.H.Wu, Y.Q.Gogolides, EvangelosNakashima, SeijiWu, YiquanGoh, Kheng LimNakayama, MasanobuWu, YupingGohs, UweNakazawa, HidekiWu, YuzhouGökce, BilalNakielski, PawelXantheas, SotirisGoldhawk, Donna E.Nam, Sang-HoXi, KaiGoldstein, Aaron S.Nam, Vu BacXi, WeixianGoltsov, AlexandrNandhakumar, IrisXiao, BoqiGomes, Ana P.Näpänkangas, RitvaXiao, PennyGómez, LuisNarani, AkashXiao, YinGomez-Flores, ReynaNarayan, RogerXiao, ZhigangGómez-García, RobertoNascimbene, RobertoXie, Rong-JunGonçalves, InêsNaso, VincenzoXu, ChunlinGong, PanNath, PradipXu, JiangtaoGoñi, IsabelNavacerrada, M.A.Xu, JinyangGonon, MauriceNavarro, Jorge A.R.Xu, XiaochuanGonzález, J.F.Naydenova, IzabelaYada, MitsunoriGonzález, SergioNazare, ShonaliYagoubi, Jalal ElGonzález-Diaz, Óscar M.Nazari, AliYahiaoui, Riad.Goodall, RussellNdu, UdonnaYahiro, HidenoriGoodpaster, JohnNebot, EnriqueYamamoto, NamikoGoppelt-Struebe, M.Nedelec, Jean-MarieYamanaka, MasamichiGorash, YevgenNelson-Cheeseman, B.B.Yamashita, KimihiroGorfman, SemënNersisyan, H.H.Yan, BangboGorria, PedroNesmelov, Yuri E.Yan, LiangGotor, Francisco J.Neto, D.M.Yan, LiboGraetz, KathrinNeves, RuiYan, QiminGraeve, Olivia A.Ng, Ka MingYanagida, AkiraGrajcar, AdamNgai, Kia L.Yáñez-Sedeño, PalomaGramlich, WilliamNguyen, GiangYang, Cheng-FuGrande, TorNguyen, Vu-HieuYang, Chung-WeiGranitzer, PetraNi, MengYang, FuqianGranville, Anthony M.Ni, NaYang, HoichangGrassl, PeterNia, Raheleh PartoviYang, HongtaGrasso, SalvatoreNiakolas, Dimitrios K.Yang, HuaGrate, JayNiazi, Nabeel KhanYang, KesongGravel, EdmondNicholson, John W.Yang, Keun-HyeokGray, Derek G.Nie, MengyanYang, LinaGraybeal, BenjaminNijmeijer, ArianYang, MijiaGreen, Daniel E.Nikiforov, AntonYang, QingyuGreenhalgh, EmileNikos, KatsarakisYang, ShoufengGreenly, Justin M.Nilius, NiklasYannick, MugnierGreer, A. LindsayNimbalkar, SanjayYao, JianGremillard, LaurentNing, HaibinYasaee, MehdiGrenfell, JamesNischang, IvoYasuhara, HideakiGresil, MatthieuNishitani, YosukeYat, WongGriesser, ThomasNishiwaki, TomoyaYavari, S. AminGrillo, FedericoNisticò, NicolaYavitt, JosephGroll, JuergenNisticò, RobertoYee, LachlanGross, Michael D.Nitschke, MirkoYeh, C.L.Grunewald, SteffenNobile, LucioYeh, Chih-koGrunwald, RüdigerNobili, StefaniaYeh, Jui-MingGu, FanNocetti, MichelaYeih, Wei-ChungGu, XiaotingNoguchi, YujiYeon, JejoonGuan, XiaoweiNolas, GeorgeYeon, Jung HeumGuelcher, Scott A.Nolte, IngoYerra, NarendranathGuemes, Jesus AlfredoNonappa, NonappaYi, LongGuénin, ErwannNorouzzadeh, PayamYildirim-Ayan, EdaGueorguiev, G.K.Norvez, SophieYildirimer, L.Guessasma, SofianeNosrati, MehdiYin, XuesongGuha, SuchiNotta-Cuvier, DelphineYokoi, ToshiyukiGulgunje, PrabhakarNovais, Rui Miguel T.Yong, KijungGulino, AntoninoNovak, RichardYoo, ByungseokGullon, PatriciaNovak, SpencerYoo, Chung-YulGuo, Gia-LuenNovio, FernandoYoon, Ho GyuGuo, WeiNowak-Michta, AnetaYoon, JonghunGuo, YuzhengNuernberger, SylviaYoon, MinjoongGupta, ManojNurrohman, HamidYoon, SeokhyunGupta, MohitNyberg, EricYoshida, HiroshiGupta, NikhilNyman, JeffryYoshida, NaokoGupta, SanjuO’Dowd, Noel P.Yoshihara, HiroshiGupta, VijaiO’Rear, EdgarYoshihiro, TsujimotoGurieva, GalinaOakey, JohnYoshinari, MasaoGustafsson, AndersObara, TomokoYoshitake, HideakiGutierrez, JunkalObata, KotaroYost, Joseph RobertGutiérrez-González, C.F.Obraztsov, AlexanderYost, Michael J.Gutmann, TorstenOchiai, TsuyoshiYou, Nam-HoGutt, BeatriceO’connor, Kevin E.Young, Derek S.Guzmán, EduardoOestreicher, AnneroseYoung, TimothyHabibzadeh, SajadOgawa, AkikoYouseffi, MansourHaddadi, FaridOgawa, TakahiroYousif, BelalHaddag, B.Oh, Daniel S.Yu, Denis Y.W.Hadimani, Ravi L.Oh, Jae-EunYu, DongHadjadj, AomarOh, Jae-MinYu, GuihuaHadjiargyrou, MichaelOh, Jung HwanYu, HailiangHadjikakou, Sotiris K.Oh, Won-ChunYu, Q.L.Hagiwara, HidehisaOhara, KeishiYu, Ruei-SungHahn, Jae RyangOhkubo, IsaoYu, WeilingHaider, WaseemOhsedo, YutakaYu, XinjunHakimi, OsnatOjard, GregYu, ZhengshanHale, MicahOkada, MichioYue, ZhongrenHall, ColinOkayasu, MitsuhiroYun, HongseokHallett, Jason P.Okesola, BabatundeŽagar, GoranHalloran, JohnOkochi, MinaZaghi, Arash E.Ham, Hyung ChulOla, O.T.Zalnezhad, ErfanHamasha, Mohammad M.Oliva, DavidZammarano, MauroHamawand, IhsanOliveira, J.P.Zandi, OmidHameed, NisharOlivier, MonginZaniewski, John P.Hamilton, CarterOmar, OmarZanutta, AlessioHamuyuni, JosephOno, KanjiZaraska, LeszekHan, BoOoyama, YousukeZea Escamilla, EdwinHan, Dong WookOpel, OliverZenk, Christopher H.Han, Jung-SukOpitz, JörgZhan, HaifeiHan, KeOpris, Dorina M.Zhang, ChaoqunHan, Min-CheolOrazi, LeonardoZhang, DayiHanaor, DorianOrlando, CalogeroZhang, FanHangai, YoshihikoOrlando, TomasZhang, FeiwuHany, HassaninOrsi, MarioZhang, FengyuanHariri-Ardebili, M.A.Ortega, J. MarcosZhang, GangHarkonen, JaakkoOrtelli, SimonaZhang, H.Harnois, MaximeOrtenzi, Marco A.Zhang, HaifeiHarris, PeterOstoja-Starzewski, MartinZhang, HedongHärtel, SebastianOtsuki, BungoZhang, LiangHarting, JensOttonello, GiulioZhang, LiyingHartwig, K.T.Otvos, LaszloZhang, LongheHasan, MainulOuisse, ThierryZhang, MengHase, AlanOzcelik, V. OngunZhang, MingzhongHashizume, MineoOzer, FusunZhang, XianghangHatakeyama, TatsukoPablos, CristinaZhang, XueqingHatzigeorgiou, George D.Pachego-Torgal, F.Zhang, YongHawk, J.A.Pack, Daniel W.Zhang, ZuhuaHawrylak, PawelPadovano, ElisaZhao, HanHayakawa, TohruPal, SudiptoZhao, HongHayami, ShinyaPalacios García, TeresaZhao, JingHayashi, K.Paladini, AlessandraZhao, RongHayashi, TakahiroPalanisamy, SureshZhao, WeifengHazlett, Linda D.Palazzetti, RobertoZhilyaev, Alexander P.He, Chao-BinPalmero, PaolaZhou, J.He, GuoweiPan, Guan-TingZhou, QiHe, JiePan, Huang HsingZhou, WeiHe, QingliangPan, Tung-MingZhou, YuanyuanHe, YouliangPan, ZhiliangZhu, HuayangHe, ZhouyingPan, ZhuZhu, YinghuaiHegemann, DirkPanagiotis, SpathisZhu, ZihuaHeidary, Damoon S.B.Panagopoulos, C.N.Zhuang, XiaoyingHeim, FredericPanchenko, IulianaZiaee, ZainabHeim, K.Pandey, G.P.Zille, AndreaHeimbs, SebastianPap, ZsoltZimmerli, WernerHeineman, William R.Papanicolaou, CatherineZinin, PavelHeinrich, GertPapanicolaou, GeorgeZitoune, RedouaneHeitkam, SaschaPapoulis, DimitriosZonta, DanieleHenaff, GilbertPaquette, Michelle M.Zornoza, EmilioHenrist, CatherineParaskevas, DimosZou, FrankHensley, Alyssa J.R.Parenti, PaoloZucca, PaoloHermann, AndreasPark, Chan-GiZucchi, FabrizioHermouet, FabienPark, Hong-GyuZuorro, AntonioHernández, SimelysPark, Hwan-Man
Hernandez Santana, M.Park, Jang-Ung


